# Transcription and replication result in distinct epigenetic marks following repression of early gene expression

**DOI:** 10.3389/fgene.2013.00140

**Published:** 2013-07-30

**Authors:** Les Kallestad, Emily Woods, Kendra Christensen, Amanda Gefroh, Lata Balakrishnan, Barry Milavetz

**Affiliations:** ^1^Department of Biochemistry and Molecular Biology, University of North Dakota School of Medicine and Health SciencesGrand Forks, ND, USA; ^2^Department of Biochemistry and Biophysics, University of Rochester School of Medicine and DentistryRochester, NY, USA

**Keywords:** simian virus 40, viral epigenetics, H3K9, H3K4, transcription, replication

## Abstract

Simian virus 40 (SV40) early transcription is repressed when the product of early transcription, T-antigen, binds to its cognate regulatory sequence, Site I, in the promoter of the SV40 minichromosome. Because SV40 minichromosomes undergo replication and transcription potentially repression could occur during active transcription or during DNA replication. Since repression is frequently epigenetically marked by the introduction of specific forms of methylated histone H3, we characterized the methylation of H3 tails during transcription and replication in wild-type SV40 minichromosomes and mutant minichromosomes which did not repress T-antigen expression. While repressed minichromosomes following replication were clearly marked with H3K9me1 and H3K4me1, minichromosomes repressed during early transcription were not similarly marked. Instead repression of early transcription was marked by a significant reduction in the level of H3K9me2. The replication dependent introduction of H3K9me1 and H3K4me1 into wild-type SV40 minichromosomes was also observed when replication was inhibited with aphidicolin. The results indicate that the histone modifications associated with repression can differ significantly depending upon whether the chromatin being repressed is undergoing transcription or replication.

## INTRODUCTION

The selective methylation of the amino terminal tails of histone H3 and H4, a well-known form of epigenetic regulation, has been associated with a number of important biological regulatory processes including the control of transcription and cellular differentiation (Atkinson et al., 2008; Corry et al., 2009; Bonasio et al., 2010; Gibney and Nolan, 2010; Lister et al., 2011; Shafa et al., 2011; Skinner, 2011). Functionally, epigenetic regulation of transcription can occur either to control a particular gene’s expression during a cell’s life, or to pass along transcriptional information following cell division. The former would be an example of intra-generational epigenetic regulation while the latter would be an example of trans-generational regulation. While both forms of regulation might occur in association with a particular gene, it has not yet been established whether the same forms of histone methylation invariably mark the chromatin of the regulated gene during intra-generational and trans-generational regulation, nor how these two forms of epigenetic regulation might be related.

Since the passing of epigenetic information from a parental cell to daughter cells during cell division is critical to transgenerational epigenetic regulation, the mechanism of this inheritance has been the subject of much interest (Abmayr and Workman, 2012). A model for the inheritance of cellular transgenerational epigenetic information has emerged in which nucleosomes containing parental epigenetic information are randomly passed to daughter DNA during replication. These nucleosomes then act to direct the modification of histones present in the newly replicated nucleosomes added to the DNA in order to conserve the parental epigenetic modifications in the daughter chromatin (Corpet and Almouzni, 2009; Zhu and Reinberg, 2011).

**FIGURE 1:**
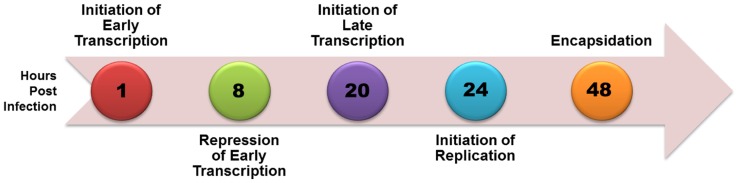
**Time course of biological processes during SV40 lytic cycle**.

Simian virus 40 (SV40), a member of the polyomavirus family, has been extensively studied as a model for eukaryotic molecular biology since its initial identification in 1960 because of its small size, organization into typical chromatin structure, and almost complete use of cellular enzymes and factors to complete its life cycle. A time course of SV40 transcription, replication, and encapsidation is shown in **Figure 1**. Upon infection the SV40 is rapidly transported to the nucleus with removal of the virus coat proteins and within 2 h early transcription begins. As the level of the major product of early transcription, T-antigen, increases it serves to repress its own expression through a feedback mechanism in which it binds to a site in the transcriptional regulatory region known as Site I. By 8 h post-infection repression of early transcription is extensive. Between 12 and 24 h post-infection late transcription and DNA replication begin with late transcription slightly preceding replication. At approximately 48 h post-infection replication is maximal. Beginning at approximately 48 h, newly replicated SV40 is bound by the products of late transcription, VP1, VP2, and VP3, to encapsidate new virus particles in a process which continues until the infected cell lyses and the newly synthesized virus is released (Acheson, 1981).

We have recently shown using a SV40 mutant which does not repress early SV40 transcription, that repression is strongly associated at late times in infection with mono-methylation of H3K9 and weakly associated at this time with mono-methylation of H3K4 (Milavetz et al., 2012). Specifically, we compared the levels of methylated H3K4 and H3K9 at 48 h post-infection in wild-type SV40 which represses early transcription and the mutant cs1085 which contains a 30-bp deletion in the regulatory region encompassing T-antigen binding Site I and does not repress early transcription (DiMaio and Nathans, 1982). We found that the percentage of SV40 minichromosomes containing H3K9me1 was reduced from 22 ± 10% in the wild-type minichromosomes to 0.66 ± 0.06% in the mutant which fails to repress. Similarly, we observed a reduction in H3K4me1 from 0.1 ± 0.07% in wild-type minichromosomes to 0.005 ± 0.007% in the mutant. In contrast, H3K4me2 went from 0.4 ± 0.3 to 0.02 ± 0.02%, H3K4me3 went from 0.08 ± 0.06 to 0.02 ± 0.02%, H3K9me2 went from 0.04 ± 0.03 to 0.17 ± 0.2%, and H3K9me3 went from 12 ± 6 to 8.2 ± 5% comparing the wild-type to the mutant. Moreover, we also showed that the changes in methylation patterns which occurred in SV40 minichromosomes during infection in mutants or following other changes in environment could also be represented in the SV40 chromatin present in virions and transferred to a subsequent infection in the viral equivalent of trans-generational epigenetic regulation (Milavetz et al., 2012). However, we do not know whether transcriptional repression occurring prior to DNA replication also results in the same effects on histone methylation. For this reason, we have extended our studies on early repression to early times in infection and characterized the changes which occur to the methylation patterns of SV40 minichromosomes. In addition, we have also investigated the role of DNA replication in introducing each of the methylated forms of H3K4 and H3K9.

## MATERIALS AND METHODS

### CELLS AND VIRUSES

Wild-type and mutant SV40 minichromosomes were prepared in the monkey kidney BSC-1 cell line (ATCC) using either wild-type 776 virus, cs1085 virus (from Dr. Daniel Nathans) or SM virus (from Dr. Chris Sullivan). The recombinants pBM129-1 and pBM131-1 were prepared in our laboratory and previously described (Hermansen et al., 1996).

### CELL CULTURE AND INFECTION

BSC-1 cells were maintained and infected as previously described with the exception of incubating cs1085 virus with the cells for 1 h, in order to increase the minichromosome yield, instead of the typical 30 min (Balakrishnan and Milavetz, 2006; Milavetz et al., 2012). SV40 minichromosomes were isolated at the indicated times post-infection as described for each of the analyses. DNA replication was inhibited with aphidicolin (final concentration 6 μM). Aphidicolin in ethanol (4 μl) was added at 24 h post-infection and minichromosomes were prepared from treated cells at 48 h post-infection.

### PREPARATION OF SV40 MINICHROMOSOMES

SV40 minichromosomes were harvested at the desired time as previously described (Balakrishnan and Milavetz, 2006; Milavetz et al., 2012) with one minor modification. After transferring the lysed cells to the 15 ml centrifuge tube, an additional 1 ml of nuclei preparation buffer was used to rinse the flask and was subsequently added to the centrifuge tube in order to maximize the yield of minichromosomes from each infection.

### CHROMATIN IMMUNOPRECIPITATION

Chromatin immunoprecipitation (ChIP) kits were obtained from Millipore and the protocol was followed as previously described (Milavetz et al., 2012). The antibodies used included: H3K4me1 (07-436, Millipore), H3K4me2 (39141, Active Motif), H3K4me3 (04-745, Millipore), H3K9me1 (ab9045, Abcam), H3K9me2 (ab1220, Abcam), H3K9me3 (ab8898, Abcam), and RNA polymerase II (RNAPII; 05-623, Millipore). All antibodies were ChIP validated by the respective vendors. Hundred microliters of protein A agarose, 800 μl of ChIP dilution buffer, and 7.5 μl of each antibody was used in a protein low-bind tube. The mixture was rotated for 5 h at 4°C on an end to end rotator in a refrigerator to bind the antibody to protein A agarose. Following binding of the antibody, the protein A agarose was spun down at 2,000 × *g* for 2 min and the supernatant discarded. Eight hundred microliters of fresh ChIP dilution buffer was added and either 100 or 200 μl of the chromatin to be analyzed was added. The samples containing antibody bound to protein A agarose and chromatin were incubated with end to end rotation for a further 7 h at 4°C. The chromatin bound to protein A agarose was washed according to the manufacturer’s protocol and eluted as previously described (Milavetz et al., 2012).

### PREPARATION OF DNA

Samples were prepared for PCR using an MP Bioscience Geneclean Spin Kit (#111101-200) with the following modifications. The glassmilk reagent (100 μl) was mixed with 100 of sample in a 1.5-ml centrifuge tube. The tube was mixed by repeated inversion at 2 min and again at 4 min of incubation. Following 5 min of room temperature incubation, the samples were centrifuged at 6,000 rpm for 30 s in a Micro One (Tomy) to pellet the glass. The supernatant was discarded and 200 μl of the wash buffer was added to the tube. While adding the wash the pipette tip was used to break up the pellet by both physically rubbing and vigorously pipetting up and down. The samples were inverted twice and centrifuged at 6,000 × *g* for 30 s to again pellet the glass. The supernatant was discarded and the pellets where dried in a vacuum for 5 min. The glass pellet with bound DNA was resuspended in 25 μl of Tris EDTA (TE) buffer.

### PCR AMPLIFICATION

DNA was amplified from the promoter region of the SV40 genome using the primers 5^′^-TTG CAA AAG CCT AGG CCT CCA AA-3^′^ and 5^′^-TGA CCT ACG AAC CTT AAC GGA GGC-3^′^ in a CFX Connect Real Time System thermal cycler (Bio-Rad) using “SSO Advanced DNA polymerase” (Bio-Rad). Immediately before use, the primers and DNase free water were added and 28 μl of the mix was used per sample. Two microliters of the resuspended glass milk in TE buffer was added per sample. Samples were amplified by PCR in triplicate with a melt curve applied afterward to ensure specific amplification. All sample preparation for PCR was done in either a Nuaire biological safety cabinet Model NU_425-400 or an AirClean 600 PCR Workstation (ISC BioExpress).

## RESULTS

**FIGURE 2:**
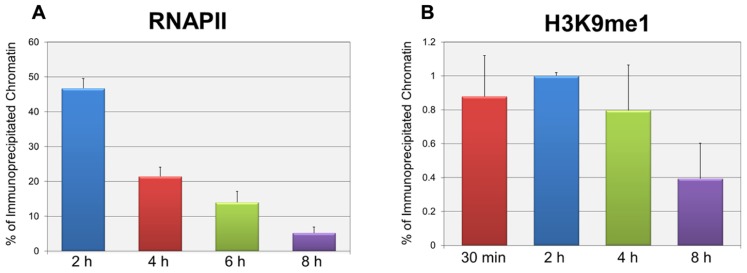
**Repression of active early transcription does not result in an increase in H3K9me1. SV40 wild-type minichromosomes were isolated between 30 min and 8 h post-infection, and subsequently subjected to ChIP analyses with antibodies to either RNA polymerase II (RNAPII) or H3K9me1.** The percentage of the input minichromosomes containing either RNAPII **(A)** or H3K9me1 **(B)** was determined by real-time PCR for each time point analyzed. All analyses were performed a minimum of three times using different preparations of SV40 minichromosomes.

In order to test whether the repression of early transcription which occurs prior to replication was also associated with the same forms of histone methylation observed when replication was occurring, we used two distinct strategies. First, we determined whether there were changes in histone methylation during the first 8 h post-infection in a wild-type infection consistent with what we previously reported for repression of early gene expression late in infection during DNA replication (Milavetz et al., 2012). We hypothesized that if transcriptional repression occurring at early times was associated with mono-methylation (me1) of H3K9 as observed during DNA replication, we would observe an increase in H3K9me1 over the first hours of an infection perhaps approaching the 20% value seen at late times when transcriptional repression was occurring. In contrast if early transcriptional repression was not associated with mono-methylation of H3K9 we would expect no effect on the levels of H3K9me1. Since we previously reported that the fraction of SV40 minichromosomes containing RNAPII decreased during the first hours of infection consistent with the repression of early transcription (Balakrishnan and Milavetz, 2006), we first confirmed that this was the case. SV40 wild-type minichromosomes were isolated 2, 4, 6, and 8 h post-infection and analyzed by ChIP for the presence of RNAPII. As shown in **Figure 2A**, we observed a slow and continual decrease in the percentage of RNAPII bound to SV40 minichromosomes between 2 and 8 h post-infection. We next determined the percentage of minichromosomes isolated at 30 min, 2, 4, and 8 h which contained H3K9me1. We did not analyze for the presence of methylated H3K4 at these times because we have previously shown that minichromosomes contain very low levels of methylated H3K4 (Milavetz et al., 2012). As shown in **Figure 2B**, we did not observe an increase in the level of H3K9me1 as expected if it was associated with transcriptional repression. H3K9me1 remained present in approximately 1% or less of the minichromosomes at this time which was similar to the level that we previously reported present in the SV40 virus particles, 2.9 ± 1% (Milavetz et al., 2012), which was used for the infection.

**FIGURE 3:**
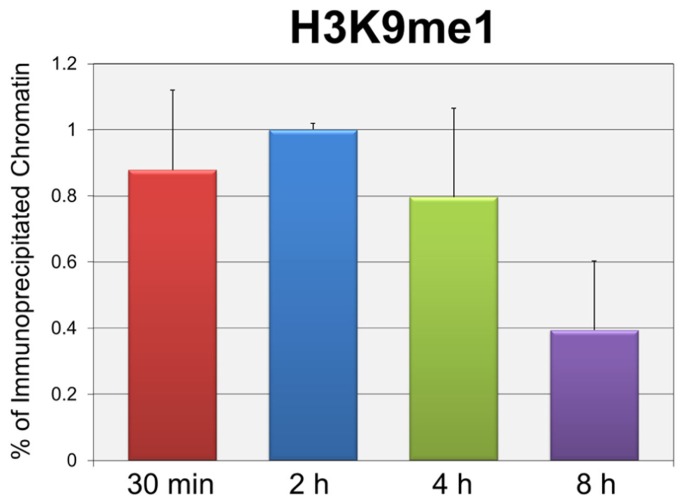
**H3K9me2 is significantly increased during active early transcription in the site I deletion mutant cs1085.** Wild-type and cs1085 SV40 minichromosomes were isolated from appropriately infected cells at 30 min and 8 h post-infection. Isolated minichromosomes were subjected to ChIP analyses with antibodies against H3K9me1, H3K9me2, and H3K9me3, and the percentage of input minichromosomes containing each form of methylated H3 determined by real-time PCR. The results are displayed as the ratio of the percentage of minichromosomes isolated at 8 h which contain a particular modification divided by the percentage of minichromosomes isolated at 30 min which contain the same modification. Ratios greater than 1 indicate that a modification is increasing during the period from 30 min to 8 h, while a ratio less than 1 indicates that the modification is decreasing during this period of infection. All analyses were performed a minimum of three times using different preparations of SV40 minichromosomes.

Secondly, we determined whether infection by the mutant cs1085 which lacks Site I and fails to repress early transcription resulted in a changed pattern of histone methylation compared to wild-type virus during the same time. Again, we focused only on the methylated forms of H3K9 at this time because we have previously shown that there is very little if any methylated H3K4 at the very early times in question (Milavetz et al., 2012). SV40 minichromosomes were prepared at the indicated times, subjected to ChIP analyses and the percentage of minichromosomes containing each methylated form of H3K9 determined by real-time PCR. The data is represented as the percentage of minichromosomes containing the modification present at 8 h of infection divided by the percentage present at 30 min of infection. A ratio less than 1 indicates that the percentage of minichromosomes carrying a particular methylated H3 is reduced over this period. As shown in **Figure 3**, we observed that for both the wild-type and cs1085 mutant we observed a reduction in the relative amount of H3K9me1 and H3K9 tri-methylation (H3K9me3) present in minichromosomes between 30 min and 8 h post-infection. However, while the amount of H3K9 di-methylation (H3K9me2) was reduced during this period in the wild-type virus, the amount was significantly increased in the cs1085 mutant. These results suggest that repression of early gene expression during active transcription occurs by a process in which the levels of H3K9me2 are kept low.

**FIGURE 4:**
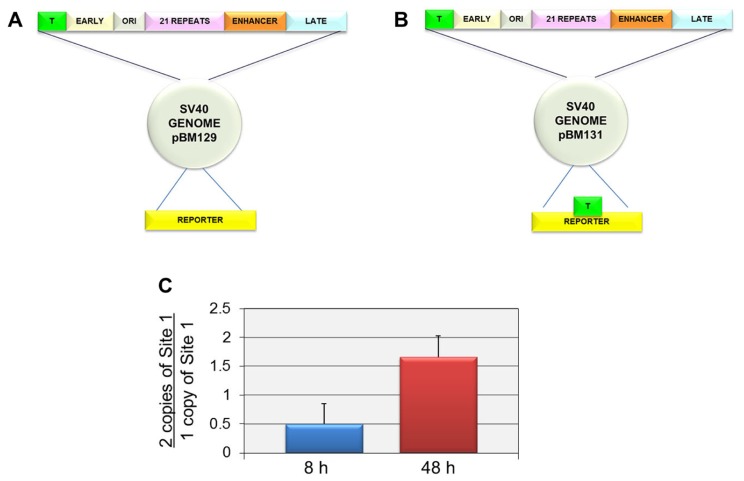
**Two copies of Site I directs the incorporation of more H3K9me1 compared to one copy of Site I in SV40 minichromosomes isolated at 48 h post-infection but not at 8 h post-infection.** SV40 minichromosomes were prepared from cells infected with pBM129-1 (one copy of Site 1) or pBM131-1 (two copies of Site 1) at 8 and 48 h post-infection. The percentage of SV40 minichromosomes containing H3K9me1 was determined by ChIP analyses for each preparation of minichromosomes at each time point followed by real-time PCR. The results are displayed as the ratio of the percentage of minichromosomes containing two copies of Site I immunoprecipitated by antibody to H3K9me1 over the corresponding percentage for minichromosomes containing one copy of Site I. A schematic of the structure of the SV40 recombinants pBM129-1 is shown in **(A)** and pBM131-1 in **(B)**. pBM131-1 contains a second copy of Site I introduced into the reporter region of the basic recombinant, pBM 129-1. The results of this analysis are shown in **(C)**. All analyses were performed a minimum of three times using different preparations of SV40 minichromosomes.

In order to independently confirm that Site I was responsible for the introduction of H3K9me1 at late times but not early times, we compared the level of H3K9me1 in an SV40 recombinant containing two copies of Site I (pBM131-1) to a parental recombinant containing only a single copy of Site I (pBM129-1). We hypothesized that if Site I was responsible for the introduction of H3K9me1 in a replication dependent manner, we would observe an increase in the percentage of H3K9me1 in the recombinant compared to the parental virus at late times but not at early times when replication was not occurring. For these studies we used recombinant viruses originally prepared to study the ability of SV40 regulatory sequences to phase nucleosomes and generate nucleosome free regions in SV40 chromatin. The parental recombinant and its construction as well as the recombinant containing two copies of Site I have been previously described (Hermansen et al., 1996). The structures of both of these constructs are shown in **Figure 3**. The parental construct pBM129-1 has a single copy of Site I in the regulatory region as in the wild-type virus (**Figure 4A**). pBM131-1 has two copies of Site I, one located as in pBM129-1 and a second copy present in the reporter region as shown in **Figure 4B**. The results of this analysis are graphically represented in **Figure 4C**. As shown at 8 h post-infection when Site I should be active down-regulating early transcription we observed a ratio of 0.50 ± 0.35 indicating that there was less methylation of H3K9me1 at this time in the recombinant carrying two copies of Site I than in the parental recombinant with only one copy. In contrast at 48 h post-infection when replication is occurring we observed a ratio of 1.66 ± 0.37 confirming that Site I is capable of directing the introduction of H3K9me1 when SV40 is replicated. Interestingly it is also apparent that the second copy of Site I can function during replication outside of its normal location within the virus genome.

**FIGURE 5:**
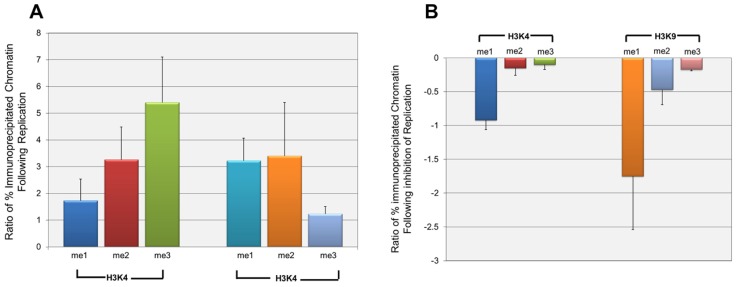
**H3K4me1 and H3K9me1 are introduced into wild-type SV40 minichromosomes primarily during active replication.** Wild-type SV40 minichromosomes were isolated at 24, 48, and 48 h post-infection following treatment with the DNA replication inhibitor aphidicolin from 24 to 48 h post-infection. The percentages of SV40 minichromosomes containing methylated H3K4 and H3K9 were determined by ChIP analyses followed by real-time PCR. The relative increase of each methylated form of H3K4 and H3K9 following DNA replication from 24 h to 48 h post-infection is shown in **(A)**. The relative increase is shown as the ratio of the fold increase of a particular form of methylated H3 between 24 and 48 h post-infection divided by the corresponding fold increase in the amount of SV40 minichromosomes between these times. Ratios greater than 1 indicate that a particular methylated form of H3 is preferentially being introduced into newly replicated minichromosomes at a rate faster than the increase in the pool size of SV40 minichromosomes. The effects of the inhibition of DNA replication from 24 to 48 h post-infection on the introduction of methylated H3K4 and H3K9 are shown in **(B)**. The results are shown as the ratio of the fold decrease in the amount of a particular form of methylated H3 in minichromosomes following inhibition of DNA replication divided by the fold decrease in minichromosomes resulting from inhibition of replication. Ratios less than or equal to 1 indicate that a particular methylated form of H3 is inhibited to a greater or the same extent as the inhibition of replication of the total SV40 minichromosomes. All analyses were performed a minimum of three times using different preparations of SV40 minichromosomes.

Since the effect of repression on H3K9me1 was only seen at late times in infection, it seemed likely that it was either directly or indirectly related to the replication of SV40 DNA which was occurring at this time. In order to test his hypothesis we determined the effect of the inhibition of replication on the introduction of methylated H3K4 and H3K9. SV40 minichromosomes were prepared at 24 h post-infection when replication was beginning and at 48 h post-infection in the presence or absence of aphidicolin, a specific inhibitor of eukaryotic DNA replication (Ohashi et al., 1978). SV40 minichromosomes were then subjected to ChIP analysis with antibodies to methylated H3K4 and H3K9. We first investigated the introduction of methylated H3 during the increase in SV40 chromatin resulting from replication between 24 and 48 h post-infection. Since we generally observe a 50- to 200-fold increase in the pool size of SV40 minichromosomes between 24 and 48 h post-infection, we compared the increase in a particular form of modification to the increase in the amount of SV40 minichromosomes. We expected that this ratio would be 1 if both the SV40 minichromosomes and form of modification were increasing at the same rate, greater than 1 if the newly replicated minichromosomes were more likely to contain the form of modification, or less than 1 if the minichromosomes were increasing faster than the introduction of the modified histone H3. The results of this analysis are graphically represented in **Figure 5A**. Based upon the observed ratios, all methylated forms of H3K4 and H3K9 were being introduced into the newly replicated minichromosomes at a rate faster than the increase in SV40 chromatin. However, H3K4me2 and H3K9me3 appeared to be introduced at rates close to the rate of increase of chromatin (1.74 and 1.23, respectively), while the other methylated forms of H3 were introduced at rates much greater than 1.

Next, we determined whether the introduction of a particular form of methylated H3 was actually dependent upon ongoing DNA replication. If ongoing DNA replication was necessary for the introduction of a particular methylated form of H3, inhibition of replication with aphidicolin should also block the introduction of the methylated form of H3. In contrast if the introduction of a methylated form of H3 was due to some other biological process, one would expect little if any effect on the introduction of the methylated form of H3 following inhibition of replication. SV40 minichromosomes were isolated from cells treated with aphidicolin from 24 to 48 h post-infection or from untreated cells at 48 h post-infection and subjected to ChIP analysis and real-time PCR. For each methylated form of H3, we then calculated the ratio of the decrease in methylated H3 to the decrease in the amount of SV40 minichromosomes following inhibition of replication. A ratio of 1 or greater would indicate that the introduction of methylated H3 was equal to or even greater than the reduction in the amount of SV40 chromatin, while a ratio near 0 would indicate that the introduction of methylated H3 was independent of DNA replication. The results of this analysis are graphically represented in **Figure 5B**. As shown in the figure the ratios for H3K9me1 (1.75) and H3K4me1 (0.92) were similar to or greater than 1 indicating that the introduction of these two methylated forms of H3 into SV40 chromatin were directly dependent upon DNA replication. The ratios for three of the methylated forms of H3 were very low including H3K4me2 (0.15), H3K4me3 (0.10), and H3K9me3 (0.17) indicating that these methylated forms of H3 were being introduced in the absence of direct DNA replication. The ratio for H3K9me2 (0.47) was intermediate between the other forms of methylated H3 suggesting that it was at least in part dependent upon replication. While we believe that the changes observed following aphidicolin treatment are primarily a result of the extensive inhibition of replication, we cannot exclude the possibility that indirect effects on transcription or induction of the DNA damage response following aphidicolin might also be contributing to changes in histone modifications.

## DISCUSSION

In SV40 minichromosomes, repression of early gene expression by T-antigen binding to Site I in the viral regulatory region was shown to result in distinct epigenetic marks at early and late times post-infection. At early times when only early transcription was occurring T-antigen binding resulted in the inhibition of the introduction of H3K9me2, while at late times when replication was occurring T-antigen binding resulted in the introduction of H3K9me1. The latter was first shown in a previous publication (Milavetz et al., 2012).

These results raise interesting questions concerning the mechanisms responsible for the introduction of epigenetic marks at the two time points in infection. Clearly, T-antigen binding is required for the introduction of the majority of H3K9me1. However, T-antigen binding does not appear to be the only signal for the introduction of H3K9me1 since a low level of H3K9me1 is still present in SV40 minichromosomes in a mutant in which T-antigen binding cannot occur. While Site I is necessary for the late introduction of H3K9me1, the Site I does not have to be located in the regulatory region since a recombinant containing an extra copy of Site I near the terminus of transcription showed an increase in H3K9me1 at late times but not early times. The location independent increase in H3K9me1 in this recombinant suggests that Site I may be functioning like an enhancer to direct epigenetic changes (Calo and Wysocka, 2013).

It seems likely that the T-antigen directed introduction of H3K9me1 is mechanistically related to DNA replication. First, we have previously shown that at late times in infection H3K9me1 was specifically associated with SV40 minichromosomes actively undergoing replication using a two-step ChIP protocol (Balakrishnan and Milavetz, 2009) in which actively replicating minichromosomes were immunologically selected for subsequent analysis using an antibody to RPA70 a replication protein (Balakrishnan and Milavetz, 2009). Second, this association was confirmed by characterizing SV40 chromatin following inhibition of replication by aphidicolin. H3K9me1 appeared to be directly related to replication since it increased when replication occurred and was completely blocked when replication was blocked. Although H3K4me1 also appeared to be a direct result of replication the other methylated forms of H3K4 and H3K9 appeared to result from post-replication maturation. The introduction of H3K9me3 following replication has been shown in HeLa cells to occur via a maturation process in which the H3K9me3 is introduced into previously replicated chromatin containing H3K9me1 (Loyola et al., 2009). It is not clear how the binding of T-antigen to Site I at early times results in the inhibition of the incorporation of H3K9me2. Potentially T-antigen might be disrupting the normal biological pathways linking H3K9me1 to H3K9me2 and H3K9me3.

These results are not consistent with a model of chromatin replication in which the pre-existing histone modifications present in the parental chromatin are duplicated in the daughter chromatin during replication (Corpet and Almouzni, 2009; Zhu and Reinberg, 2011). Instead these results suggest that in SV40 minichromosomes DNA replication can serve as an epigenetic switch in which newly replicated chromatin can be epigenetically modified in response to specific signals such as T-antigen binding to Site I. It seems unlikely that the H3K9me1 present during replication is simply a consequence of H3K9me1 being present in parental chromatin. If this were the case one would expect similar levels of H3K9me1 in both the cs1085 mutant and the wild-type virus since both contain H3K9me1 at early times. Secondly, a model in which pre-existing H3K9me1 drives the introduction of H3K9me1 following replication does not fit with the data obtained with the recombinant containing an extra copy of Site I. At early times the recombinant and its parental strain both contain similar levels of H3K9me1 yet at late times there is a significant increase in the amount of H3K9me1 present in replicated minichromosomes. This epigenetic switching hypothesis is consistent with a recent publication showing that replication of *Drosophila* chromatin occurs through a process in which pre-existing histone modifications are lost at the replication fork and histone modifications are re-introduced following replication by modifying complexes which remain closely associated with the replicating chromatin (Petruk et al., 2012). The results differ in that in the publication pre-existing modifying complexes are thought to drive the introduction of post-replicative histone modifications while in SV40 the post-replicative changes are driven by the binding of the repressive factor T-antigen.

The most likely reason for the epigenetic switch is to ensure that newly replicated minichromosomes are not capable of activation for early transcription at late times in infection. Allowing activation of early transcription as in the case of the mutant cs1085 has been shown to result in a significant reduction in the pool size of SV40 minichromosomes and yield of virus late in infection (Milavetz et al., 2012). This epigenetic switch may also play a critical role in controlling the relative pool sizes of transcribing, replicating, and encapsidating SV40 minichromosomes.

While an epigenetic switch associated with replication appears to have a biological relevance for SV40 it is not yet clear whether a similar process functions in cellular chromatin. However, it is interesting to speculate that a similar process could act during cellular differentiation to prepare newly replicated chromatin for subsequent activation or repression of transcription in response to specific signals introduced during replication as part of the differentiation pathway.

## Conflict of Interest Statement

The authors declare that the research was conducted in the absence of any commercial or financial relationships that could be construed as a potential conflict of interest.
