# Erratum: Transcription and replication result in distinct epigenetic marks following repression of early gene expression

**DOI:** 10.3389/fgene.2013.00259

**Published:** 2013-12-02

**Authors:** Barry Milavetz, Les Kallestad, Emily Woods, Kendra Christensen, Amanda Gefroh, Lata Balakrishnan

**Affiliations:** ^1^Department of Biochemistry and Molecular Biology, University of North Dakota School of Medicine and Health SciencesGrand Forks, ND, USA; ^2^Department of Biochemistry and Biophysics, University of Rochester School of Medicine and DentistryRochester, NY, USA

**Keywords:** viral epigenetics, SV40, transcription, replication origin, H3K9, H3K4

In reviewing our recent publication, “Transcription and replication result in distinct epigenetic marks following repression of early gene expression” published 30 July 2013, we noted that there was an error in Figure 3. Apparently between the original submission and our review of the proofs we did not notice that the correct Figure 3 graphic had been replaced by a copy of the graphic in Figure 2B. We regret the error.

**Figure 3:**
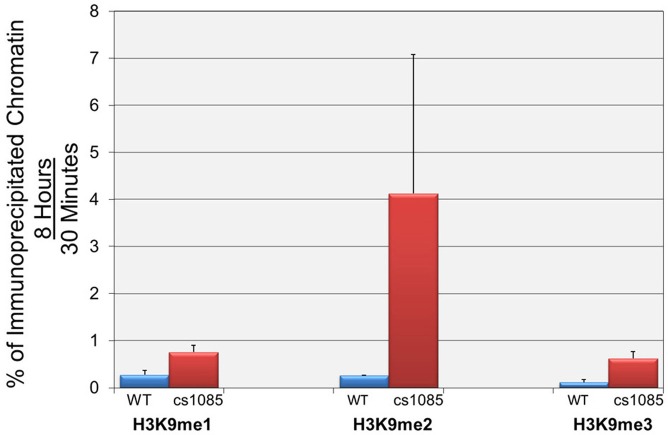
**H3K9me2 is significantly increased during active early transcription in the site I deletion mutant cs1085.** Wild-type and cs1085 SV40 minichromosomes were isolated from appropriately infected cells at 30 min and 8 h post-infection. Isolated minichromosomes were subjected to ChIP analyses with antibodies against H3K9me1, H3K9me2, and H3K9me3, and the percentage of input minichromosomes containing each form of methylated H3 determined by real-time PCR. The results are displayed as the ratio of the percentage of minichromosomes isolated at 8 h which contain a particular modification divided by the percentage of minichromosomes isolated at 30 min which contain the same modification. Ratios greater than 1 indicate that a modification is increasing during the period from 30 min to 8 h, while a ratio less than 1 indicates that the modification is decreasing during this period of infection. All analyses were performed a minimum of three times using different preparations of SV40 minichromosomes.

